# Accessing orthographic representations from speech: The role of left ventral occipitotemporal cortex in spelling

**DOI:** 10.1002/hbm.22709

**Published:** 2014-12-12

**Authors:** Philipp Ludersdorfer, Martin Kronbichler, Heinz Wimmer

**Affiliations:** ^1^ Centre for Cognitive Neuroscience University of Salzburg Salzburg Austria; ^2^ Neuroscience Institute Christian‐Doppler‐Clinic, Paracelsus Medical University Salzburg Austria

**Keywords:** fMRI, neuroimaging, spelling, orthographic representations, ventral occipitotemporal cortex

## Abstract

The present fMRI study used a spelling task to investigate the hypothesis that the left ventral occipitotemporal cortex (vOT) hosts neuronal representations of whole written words. Such an orthographic word lexicon is posited by cognitive dual‐route theories of reading and spelling. In the scanner, participants performed a spelling task in which they had to indicate if a visually presented letter is present in the written form of an auditorily presented word. The main experimental manipulation distinguished between an orthographic word spelling condition in which correct spelling decisions had to be based on orthographic whole‐word representations, a word spelling condition in which reliance on orthographic whole‐word representations was optional and a phonological pseudoword spelling condition in which no reliance on such representations was possible. To evaluate spelling‐specific activations the spelling conditions were contrasted with control conditions that also presented auditory words and pseudowords, but participants had to indicate if a visually presented letter corresponded to the gender of the speaker. We identified a left vOT cluster activated for the critical orthographic word spelling condition relative to both the control condition and the phonological pseudoword spelling condition. Our results suggest that activation of left vOT during spelling can be attributed to the retrieval of orthographic whole‐word representations and, thus, support the position that the left vOT potentially represents the neuronal equivalent of the cognitive orthographic word lexicon. *Hum Brain Mapp, 36:1393–1406, 2015*. © **2014 The Authors Human Brain Mapping Published by Wiley Periodicals, Inc.**

## INTRODUCTION

One of the issues in the cognitive and neuroscientific study of reading processes concerns the existence of an orthographic word lexicon, that is, a memory system containing representations of the exact letter sequences of all known written words. Such orthographic whole‐word representations are assumed by cognitive dual‐route models of word reading [Coltheart et al., 2001; Perry et al., 2007]. These models distinguish between a visual‐lexical route from print to sound mediated by the orthographic lexicon (for known written words) and a sublexical‐phonological route via serial grapheme‐phoneme coding (for unknown written words). In contrast, the assumption of an orthographic word lexicon is explicitly denied by connectionist (single route) learning models of reading which demonstrated that complex adjustment of connection weights between distributed representations of letters, hidden units, and phonemes allow correct reading even of irregular words [Harm and Seidenberg, 2004; Plaut et al., 1996].

In neuroimaging research of word reading, the assumption of an orthographic word lexicon has also been controversial. Based on frequent failures to identify a visual region with higher activation for written words relative to pseudowords, Jobard et al. [2003] concluded that there is no brain region equivalent to a written word lexicon. Nevertheless, one brain region has received specific interest with respect to neuronal representations of written words, namely, the left ventral occipitotemporal cortex (vOT). This region, situated between ventral occipital and temporal lobe, on the border of posterior fusiform and inferior temporal gyrus (ITG) has been shown to be reliably activated by visually presented words [Jobard et al., 2003; Mechelli et al., 2003; Turkeltaub et al., 2002]. The two most prominent accounts of left vOT functioning, however, stand in conflict with the idea of orthographic whole‐word representations. The visual word form area (VWFA) hypothesis of Dehaene and Cohen (e.g., 2011) postulates that a left vOT region (termed the VWFA) is specifically engaged by visual word processing and hosts neurons tuned to orthographic features. However, in contrast to the orthographic word lexicon assumption, these neuronal representations are assumed to be sublexical (i.e., representations of recurring letter sequences within words; Dehaene et al., 2002]. The interactive account of vOT functioning by Price and Devlin [2011]—inspired by connectionist reading models—suggests that left vOT is an interface area linking generic visual input to higher‐level associations such as phonology and meaning. This account explicitly denies experience‐driven neuronal representations of whole words or sublexical orthographic features in left vOT.

Our research group provided findings consistent with the orthographic word lexicon assumption showing that during reading the left vOT is not only engaged in sublexical letter string computations [e.g., Dehaene et al., 2002] but also in orthographic whole‐word coding. In Kronbichler et al. [2004], we interpreted an inverse relation between written word frequency and left vOT activation as reflecting easier access to frequently relative to rarely used orthographic whole‐word representations. In Kronbichler et al. [2007, 2009], we contrasted familiar and unfamiliar spellings for the same phonological words (e.g., TAXI vs. TAKSI) and found decreased activation in left vOT for the familiar spellings. While the reduced activation for familiar words presumably reflected activation of a single whole‐word representation, the high activation for unfamiliar letter strings may have resulted from activation of several sublexical representations (i.e., letters or letter sequences; Kronbichler et al., 2007]. Similar inverse effects of orthographic familiarity on left vOT activation (i.e., words < pseudohomophones and pseudowords) were found in several other studies [Bruno et al., 2008; Twomey et al., 2011; van der Mark et al., 2009]. In Schurz et al. [2010], we found that the length of familiar words did not affect left vOT activation whereas the length of pseudowords led to a marked length effect. Absence of a length effect for words is expected when orthographic word representations allow for whole‐word recognition. Further support for orthographic word representations in left vOT came from an fMRI adaptation study by Glezer et al. [2009]. The authors showed that the strong adaptation (= reduced activation) effect in vOT present for word repetition priming (coat–coat) disappeared when the prime differed in just one letter from the target word (boat–coat). This strong selectivity for words was absent for pseudowords for which the change of one letter from prime to target still led to adaptation.

Also of interest for the hypothesis of orthographic whole‐word representations in left vOT are neuroimaging studies presenting spoken words in the context of spelling or writing tasks. In these studies, orthographic information (i.e., the spelling) has to be accessed in the absence of visual input. Two recent meta‐analyses of neuroimaging studies of spelling and writing [Purcell et al., 2011a; Planton et al., 2013] identified consistent activation clusters in left vOT that closely correspond to left vOT clusters identified by neuroimaging studies of word reading. Importantly, two of the spelling studies included in the meta‐analyses directly showed that there is overlapping left vOT activation for word reading and word spelling [Rapp and Lipka, 2011; Purcell et al., 2011b]. However, critical for the orthographic word lexicon assumption, none of the previous spelling/writing studies assured reliance on orthographic whole‐word representations and spellings could have also been derived via sublexical processes (i.e., phoneme–grapheme conversions).

The present German‐based fMRI study is the first to investigate whether left vOT activation during spelling reflects the retrieval of orthographic whole‐word representations. To avoid complex manual writing or letter naming responses we relied on the elegant spelling probe task of Rapp and Lipka [2011] in which participants have to indicate with a button press if a visual probe letter is present in the spelling of an auditorily presented word. In the context of this task we realized two word spelling conditions in which reliance on orthographic whole‐word representations was either assured or optional and a pseudoword spelling condition in which no reliance on such representations was possible. To isolate brain activation related to spelling processes the spelling conditions were contrasted with control conditions that presented the same spoken words or pseudowords, but participants had to indicate if the visually presented letter corresponded to the gender of the speaker.

In the critical orthographic word spelling condition, correct spelling decisions had to be based on orthographic whole‐word representations. This was achieved by assuring that the trials of this condition were set up in a way that orthographic spelling decisions were always opposite to decisions based on phonological information (i.e., phoneme‐letter correspondences). For example, the spoken word /ti:m/ associated with the spelling TEAM (a highly familiar English loanword in German) was presented with the probe letters A or I. Correct decisions based on the orthographic whole‐word representation resulted in a “yes” response to the letter A and a “no” response to I. In contrast, phonological decisions based on the phonemes associated with the probe letters would have resulted in opposite (and incorrect) responses as /a:/ the phoneme for the letter A in German is not present in /ti:m/ whereas /i:/ the phoneme for the letter I is present. Based on our previous reading‐based findings showing that left vOT is involved in orthographic whole‐word coding [e.g., Kronbichler et al., 2007] we expected the enforced reliance on orthographic word representations in this spelling condition to result in substantial left vOT activation.

In a second word spelling condition, the orthographic‐phonological condition each trial was set up such that decisions based on orthographic whole‐word representations did not conflict with phonological decisions. For example, /ti:m/ was presented with the probe letter M. The “yes” response resulting from retrieving the orthographic word representation TEAM does not conflict with the phonological decision based on hearing the phoneme /m/ associated with the letter M in /ti:m/. This condition is comparable to conventional writing or spelling of known words with phonological information (i.e., phoneme‐letter correspondences) in addition to orthographic information. This condition—different from the orthographic word spelling condition—did not enforce reliance on orthographic whole‐word representations. However, reliance on phonological decisions was discouraged as the trials of both the orthographic and the orthographic‐phonological word spelling condition were presented with the same instruction, that is, to evaluate the presence of the probe letter in the spelling of an existing word. The main reason for adding this condition was the concern that the conflict between orthographic and phonological spelling decisions in the orthographic word spelling condition might result in increased activation in left frontal regions associated with conflict resolution [e.g., Brass et al., 2005] and potentially also in the critical left vOT region.

The third spelling condition—referred to as phonological condition—presented pseudowords instead of words in the context of the spelling probe task (Table 1 provides exemplary trials for this and the two word spelling conditions). Therefore, reliance on orthographic word representations was not possible. Instead, participants had to rely on sublexical‐phonological spelling information (i.e., phoneme‐letter correspondences). Specifically, we expected that participants might mentally assemble the spelling via serial phoneme‐to‐letter conversions and evaluate whether the visual probe letter is present in the generated letter string. The assembly of the letter string corresponding to an auditory pseudoword might activate several sublexical representations (i.e., letters and letter sequences), which should result in similar or higher left vOT activation than the activation of a single orthographic whole‐word representation for an existing word. This expectation is based on the hypothesis that spelling findings correspond to the mentioned reading‐based findings which speak for a left vOT engagement in both whole‐word and sublexical orthographic coding [Schurz et al., 2010]. However, there is also neuroimaging evidence suggesting that sublexical‐phonological spelling processes primarily rely on left superior temporal and inferior frontal regions rather than left vOT [Beeson and Rapcsak, 2003 and Omura et al., 2004]. In addition, results from lesion studies suggest that while sublexical spelling critically depends on left superior temporal regions it does not depend on left vOT [e.g., Henry et al., 2007; Philipose et al., 2007]. If this is the case, then one could expect absent vOT activation for the phonological pseudoword spelling condition.

**Table 1:** Example stimuli for the spelling conditions

Spelling condition	Auditory word/pseudoword (spelling)		Visual probe letter (associated phoneme)		Correct response
Orthographic Word	/fa:zə/	(PHASE)	p	(/p/)	Yes
	/taksi/	(TAXI)	k	(/k/)	No
Orthographic‐phonological Word	/fa:zə/	(PHASE)	a	(/a:/)	Yes
	/taksi/	(TAXI)	r	(/r/)	No
Phonological Pseudoword	/geran/	(GERAN)	e	(/e:/)	Yes
	/tiska/	(TISKA)	m	(/m/)	No

## MATERIALS AND METHODS

### Participants

Twenty‐four German‐speaking university students (15 female) participated in the present fMRI study. All participants were right‐handed, had normal or corrected‐to‐normal vision and reported no history of neurological disease or reading/spelling difficulties. Two participants had to be excluded from analysis due to excessive head motion in the scanner (>3 mm) and one participant was excluded due to technical problems during image acquisition. The age of the remaining 21 participants (14 female) ranged from 18 to 38 years (*M* = 25.1 years, *SD* = 5.0 years). All gave written informed consent and were paid for participation. Experimental procedures were approved by the Ethics Committee of the University of Salzburg.

### Tasks and Procedure

The experimental design consisted of five conditions: the orthographic, the orthographic‐phonological and the phonological spelling condition as well as the word and the pseudoword control condition. In the spelling conditions, participants had to indicate with a button press if a visually presented letter was present in the spelling of an auditorily presented word (orthographic and orthographic‐phonological condition) or pseudoword (phonological condition). Table 1 presents example stimuli for all three spelling conditions (and a detailed description is provided in the Introduction). In the control conditions, participants had to indicate if the visually presented letter (always either m for maennlich [male] or w for weiblich [female]) corresponded to the gender of the speaker of the auditory word/pseudoword. This control task was designed to control for sensory (i.e., presentation of an auditory word followed by a visual letter) and motor components (i.e., yes/no button press response) of the spelling task.

**Figure 1:**
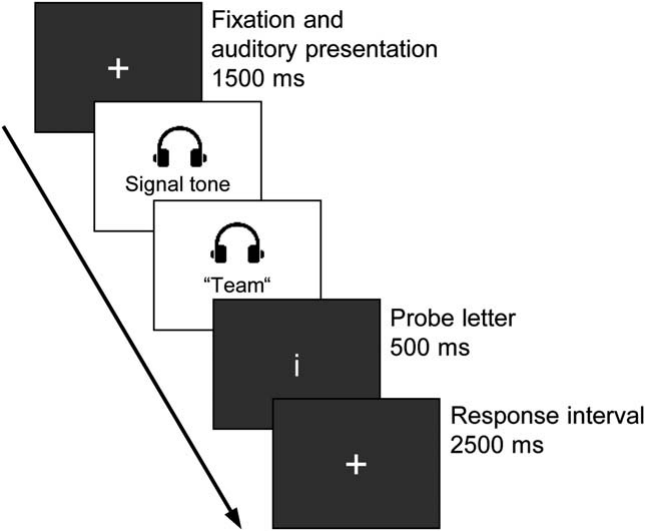
Sequence of events in a trial (for all experimental conditions). See text for timing details of auditory presentation.

The experimental conditions were presented in six blocks of five trials. Each block started with an instruction screen (1,500 ms) that indicated the task (i.e., spelling or gender decision) and informed about the stimulus type (i.e., words or pseudowords). The individual trials of all conditions began with a fixation cross that was presented centrally on the screen for 1,500 ms. During this time interval, a signal tone (200 ms) followed by an auditory word or pseudoword were presented via headphones. The length of the auditory words/pseudowords ranged from 534 to 1,068 ms (*M* = 757 ms). The end of the auditory stimuli was aligned to the end of the fixation period. After the auditory stimuli a lower‐case letter was presented centrally on the screen for 500 ms. Trials ended with a 2,500 ms response interval during which a fixation cross was presented. The sequence of events in a trial is illustrated in Figure 1. The total length of a trial was 4 s resulting in a total block length of 21.5 s. Throughout each block, visual task reminders were present centrally in the upper half of the display: a tick for the spelling probe task and the Venus symbol for the gender decision control task. The number of “yes” responses for the five trials per block varied from one to four. The experimental design also included six rest blocks (fixation periods) of the same length as the task blocks. All blocks were presented within a single run. The order of block presentation was optimized using a genetic algorithm [Wager and Nichols, 2003]. When a task block was not followed by a rest block, a short fixation period of 3,000 ms was inserted between task blocks. The total length of the experiment was approximately 14 min.

Participants were familiarized with both the spelling and the control task outside the scanner. For all conditions, we assured that participants reached >90% accuracy in the training. Auditory stimuli were presented via MR‐compatible headphones and visual stimuli were presented via a mirror on a MR‐compatible LCD screen (NordicNeuroLab, Bergen, Norway) in a white font on a dark gray background. An MR‐compatible response box was used for the participants to respond. Participants were instructed to respond with the index (yes) and middle finger (no) of their right hand. Stimulus delivery and response registration were controlled by Presentation (Neurobehavioral Systems, Albany, CA).

### Stimuli

For the word conditions of the present study (orthographic spelling, orthographic‐phonological spelling, and word control condition) 90 German nouns were selected and divided into three lists of 30 items each. Across participants, each list was used about equally often for each of the three word conditions. The lists were matched for syllable and letter length, lexical frequency, bigram frequency, and orthographic distance [orthographic Levenshtein distance 20; Yarkoni et al., 2008]. 1To measure orthographic neighborhood size we used the orthographic Levenshtein distance 20 (OLD20; Yarkoni et al., 2008) which computes the average number of insertions, deletions or substitutions from a target word to its 20 closest neighbors. In contrast to the classic orthographic neighborhood measure (ON; Coltheart et al., 1977) neighboring words do not have to have the same length as the target word. Yarkoni et al. (2008) showed that compared to the ON the OLD20 was significantly better in predicting lexical decision and pronunciation outcome. All item characteristics were based on the SUBTLEX database for German [Brysbaert et al., 2011].

A main criterion for the selection of the word items was that the words could be used in the orthographic spelling condition in either a “yes” or a “no” trial. For “yes” trials (i.e., the probe letter is present in the spelling), a typical case was that the words included a letter that is “silent” in the pronunciation of the word (e.g., the T in GOURMET or the H in KOHLE [coal]). In other cases, the words included a letter that is ambiguous with respect to associated phonemes (e.g., V is pronounced /f/ in VATER [father] but /v/ in VASE). Examples for “yes” trials with ambiguous letters are V following VISION (pronounced similarly as in English) or C following CELLO (pronounced as in English). In still other cases, the words included a letter that is highly associated with a specific phoneme (e.g., G associated with /g/), but is pronounced differently in the pronunciation of the selected word as in the case of REGIME (pronounced as in English). For “no” trials, the pronunciation of the words had to contain a phoneme highly associated with a letter that is not part of the spelling (e.g., the K in TAXI, the O in PLATEAU, or the W in VANILLE [vanilla]). As evident from the examples, a number of the selected words were of Greek, Latin or French origin and the spellings of these words could be characterized as unusual with respect to common phoneme–grapheme rules of German. However, one should note that German—similar to many other orthographies—exhibits an asymmetric regularity, that is, much less regularity in the spelling than in the reading direction. To illustrate, the long vowel /a:/ is spelled differently in SAAL (hall) and WAHL (vote). In the reading direction, however, each of the two vowel spellings is regular.

For the pseudoword conditions (phonological spelling and pseudoword control) 60 pseudowords (i.e., pronouncable nonwords) were generated and separated into two lists. Across participants, the two lists, were used equally often in the phonological spelling and in the pseudoword control condition. The lists of pseudowords corresponded to the word lists with respect to letter and syllable length, orthographic distance, and bigram frequency (see Table 2 for mean item characteristics of words and pseudowords). In addition, we took care that the pseudowords were not too similar to existing words and that the probe letters allowed a rather definite decision on whether it was included in a possible spelling of the pseudoword.

**Table 2:** Mean item characteristics

	Words	Pseudowords
Number of syllables	2.2 (0.6)	2.3 (0.5)
Number of letters	6.1 (1.2)	6.1 (1.0)
Frequency (per million)	12.6 (19.8)	–
Summated bigram frequency	54,076 (33,736)	53,974 (31,976)
Orthographic distance	2.0 (0.6)	2.0 (0.5)

Standard deviations are given in parentheses

Each of the word and pseudoword lists required an equal number of “yes” and “no” responses and the position of the probe letters requiring a “yes” response was matched across lists. Additionally, each of the words and pseudowords was recorded with a male and a female voice. Across participants, we assured that the male and female versions were presented equally often in each condition. With respect to the control conditions, we further assured that, across participants, each word was presented equally often with the corresponding “probe” letter (i.e., m for maennlich [male] and w for weiblich [female]) and with the incorrect “probe” letter.

### fMRI Data Acquisition and Analysis

A Magnetom Trio 3 Tesla Scanner (Siemens, Erlangen, Germany) was used for both functional and anatomical MR imaging. In the functional run, 396 images sensitive to blood oxygenation level dependent (BOLD) contrast were acquired with a T2*‐weighted echo‐planar imaging sequence (flip angle = 70°, TR = 2250 ms, TE = 30 ms, field of view = 210 mm, 64 × 64 matrix) using a 12 channel head coil. Thirty‐six descending axial slices (thickness = 3.0 mm; interslice gap = 0.3 mm) were acquired within each TR. Additionally, a gradient echo field map (TR = 448 ms, TE 1 = 4.49 ms, TE 2 = 6.95 ms) and a high‐resolution (1 × 1 × 1.2 mm) structural scan with a T1‐weighted MPRAGE sequence were acquired.

For preprocessing and statistical analysis, the SPM8 software was used (http://www.fil.ion.ucl.ac.uk/spm) running in a MATLAB 7.6 environment (Mathworks, Natick, MA, USA). Functional images were realigned, unwarped, corrected for geometric distortions by use of the FieldMap toolbox, and slice‐time corrected. The high‐resolution structural image was preprocessed and normalized using the VBM8 toolbox (http://dbm.neuro.uni-jena.de/vbm8). The structural image was segmented into gray matter, white matter and CSF, denoised, and warped into Montreal Neurological Institute (MNI) standard space by registering it to the DARTEL template of the VBM8 toolbox using the high‐dimensional DARTEL registration algorithm [Ashburner, 2007]. Based on these steps, a skull‐stripped version of the structural image was created in native space. The functional images were coregistered to the skull‐stripped structural image and then the parameters from the DARTEL registration were used to normalize the functional images to the MNI space. The functional images were further resampled to isotropic 3 × 3 × 3 mm voxels and smoothed with an 8 mm FWHM (Full width half maximum) Gaussian kernel.

Statistical analysis of the fMRI data was performed within a two‐stage mixed effects model. On the individual level, regression coefficients were derived for all spelling and control conditions by convolving a boxcar function with a synthetic hemodynamic response function marking the temporal position of each block. Additionally, six covariates corresponding to the motion‐correction parameters (rotations and translations) were included. The functional data in these first‐level models were high‐pass filtered with a cutoff of 128 s and corrected for autocorrelation by an AR(1) model [Friston et al., 2002]. In the first level models, the parameter estimates reflecting signal change for all conditions relative to baseline (which consisted of the rest blocks and the short fixation periods between task blocks) were calculated in the context of a General Linear Model [Henson, 2004]. The participant‐specific images were then used for the second‐level random effects analysis, which allows generalization to the population. For statistical comparisons we used a voxelwise threshold of *P* < 0.001 with an additional cluster extent threshold of *P* < 0.05, corrected for family‐wise error (FWE). For regions of interest, contrast parameter estimates (for each condition versus rest) were extracted using the MarsBar Toolbox for SPM [Brett et al., 2002]. Anatomical descriptions for activation peaks are based on the probabilistic Harvard–Oxford Atlas [Desikan et al., 2006] as implemented in FSL (http://www.fmrib.ox.ac.uk/fsl), thresholded at 25%.

## RESULTS

### Behavioral Results

As can be seen in Table 3, the spelling conditions were generally more difficult than the control conditions. This is evident from prolonged decision latencies and a higher proportion of erroneous responses (all *p*s < 0.001). There was no difference between word and pseudoword control condition in decision latencies or accuracy (*t*s < 1). Furthermore, Table 3 shows that there was no difference in decision latencies (*t*s < 1) between the spelling conditions. However, participants made more errors in the orthographic and in the phonological spelling condition compared to the orthographic‐phonological spelling condition (*p*s < 0.001).

**Table 3:** Behavioral results

	Decision latencies (ms.)	Errors (%)
Spelling conditions		
Orthographic Word	1087 (240)	18.7 (11.8)
Orthographic‐phonological Word	1053 (211)	11.3 (6.4)
Phonological Pseudoword	1073 (211)	21.1 (8.3)
Control conditions		
Word	845 (189)	4.0 (6.2)
Pseudoword	828 (213)	1.3 (2.5)

Mean decision latencies and percentage of erroneous responses are presented for the spelling and control conditions. Standard deviations are given in parentheses

### fMRI Results

#### Spelling versus Control Conditions

**Figure 2:**
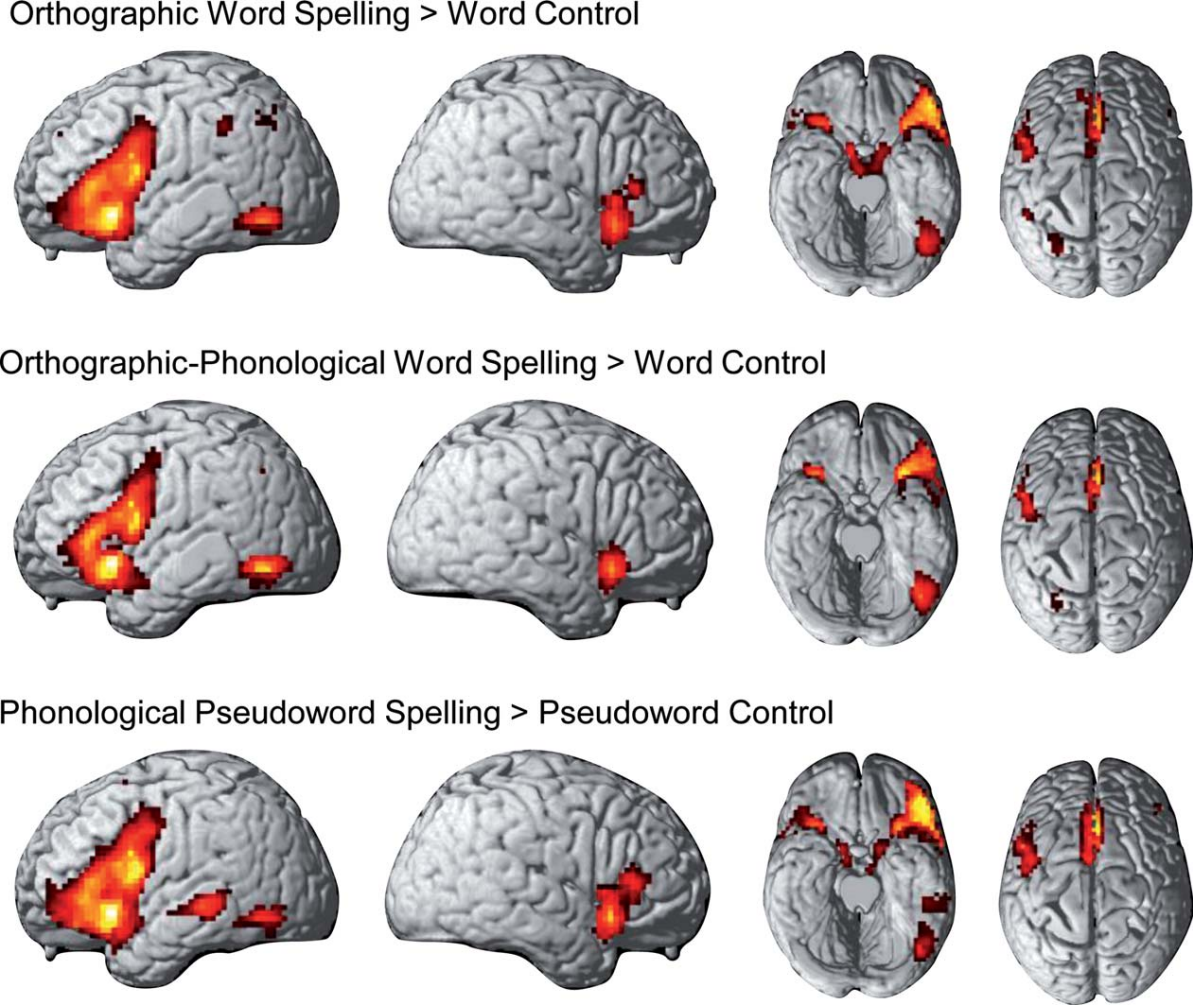
Spelling versus control conditions. Brain regions activated by the orthographic, the orthographic‐phonological, or the phonological spelling condition relative to their respective control condition. All comparisons are thresholded at *P* < 0.001, voxelwise, with an additional cluster extent threshold of *P* < 0.05, FWE corrected.

To investigate brain regions engaged by the spelling conditions we contrasted each spelling condition with its respective control condition. Specifically, the orthographic and the orthographic‐phonological spelling condition were compared to the word control condition and the phonological spelling condition was compared to the pseudoword control condition. The results of these comparisons are presented in Table 4 and Figure 2.

All three spelling conditions elicited widespread activations in left lateral frontal regions including inferior frontal gyrus (IFG), precentral gyrus, and the insular cortex. The activation maxima for the word spelling conditions were situated in the insular cortex whereas the maximum for the pseudoword spelling condition was located in the precentral gyrus. In all three spelling conditions, we also identified activations in medial frontal regions (anterior cingulate cortex/paracingulate gyrus) and in right hemispheric orbitofrontal gyrus/insular cortex. Of specific interest for our hypotheses were findings in left vOT. Here, we identified a cluster at MNI coordinates [−45 −64 −11] that exhibited activation for all spelling conditions relative to the control conditions.

**Table 4:** Spelling versus control conditions

			MNI co ordinates			
Region	H	k	*x*	*y*	*z*	*t*
*Orthographic word spelling > word contro*l						
Lateral frontal	L	1520				
Insular cortex			−30	17	−8	10.18
Inferior frontal gyrus (pTri)			−45	29	10	9.15
Precentral gyrus/Inferior frontal gyrus (pOp)			−45	5	19	8.70
Paracingulate gyrus/Anterior cingulate gyrus	L/R	660	−3	20	40	7.35
Insular cortex	R	289	36	17	−11	8.48
Lateral occipital cortex/Superior parietal lobule	L	175	−24	−64	37	5.64
Ventral occipitotemporal cortex	L	139	−45	−64	−11	8.22
Subcortical						
Caudate/Pallidum	R	304	12	5	−2	5.38
*Orthographic‐phonological word spelling > word control*						
Lateral frontal	L	1191				
Insular cortex			−30	17	−8	9.21
Precentral gyrus			−42	2	22	8.98
Inferior frontal gyrus (pTri)			−45	32	10	7.87
Paracingulate cortex / Anterior cingulate gyrus	L/R	455	−3	14	52	6.76
Frontal orbital cortex	R	164	33	20	−11	7.11
Lateral occipital cortex / Superior parietal lobule	L	127	−24	−67	34	5.96
Ventral occipitotemporal cortex	L	162	−45	−64	−11	8.72
*Phonological pseudoword spelling > pseudoword control*						
Lateral frontal	L	1691				
Precentral gyrus/Inferior frontal gyrus (pOp)			−48	5	16	9.20
Insular cortex			−30	17	−8	8.83
Frontal orbital cortex			−39	20	−8	8.19
Paracingulate gyrus/Anterior cingulate gyrus	L/R	701	−3	14	52	7.54
Frontal orbital cortex	R	348	33	20	−11	7.28
Middle temporal gyrus/Superior temporal gyrus	L	139	−48	−34	−5	6.14
Ventral occipitotemporal cortex	L	89	−45	−64	−11	5.90
Subcortical						
Pallidum/Putamen	L	87	−18	5	−2	4.79
Caudate/Pallidum	R	79	15	−1	−5	5.43

Brain regions activated by the orthographic, orthographic‐phonological or phonological spelling condition relative to their respective control condition (voxelwise threshold: *P* < 0.001, cluster extent threshold: *P* < 0.05, FWE corrected). Abbreviations: pTri = pars triangularis, pOp = pars opercularis, H = Hemisphere, k = cluster extent in voxel.

In occipitoparietal regions only the word spelling conditions evoked activation in a cluster with a peak at the border of left lateral occipital cortex and left superior parietal lobule. This cluster did not extend into the angular gyrus. When lowering the threshold to *P* < 0.001, uncorrected, also the pseudoword spelling condition led to a similar occipitoparietal cluster with a peak at [−42 −55 49] (peak *t* value = 4.06; cluster extent = 32 voxels). In left superior and middle temporal gyrus (STG and MTG, respectively) only the pseudoword spelling condition elicited activation relative to the pseudoword control condition. Even with a lowered threshold (*P* < 0.001, uncorrected) there was no activation for the word spelling conditions compared to the word control condition. We want to note, however, that relative to rest all spelling conditions elicited marked activation in bilateral STG (see Supporting Information Results). We additionally identified subcortical regions in the caudate/pallidum to be activated by the orthographic and the phonological spelling condition.

#### Comparisons among the Spelling Conditions

**Figure 3:**
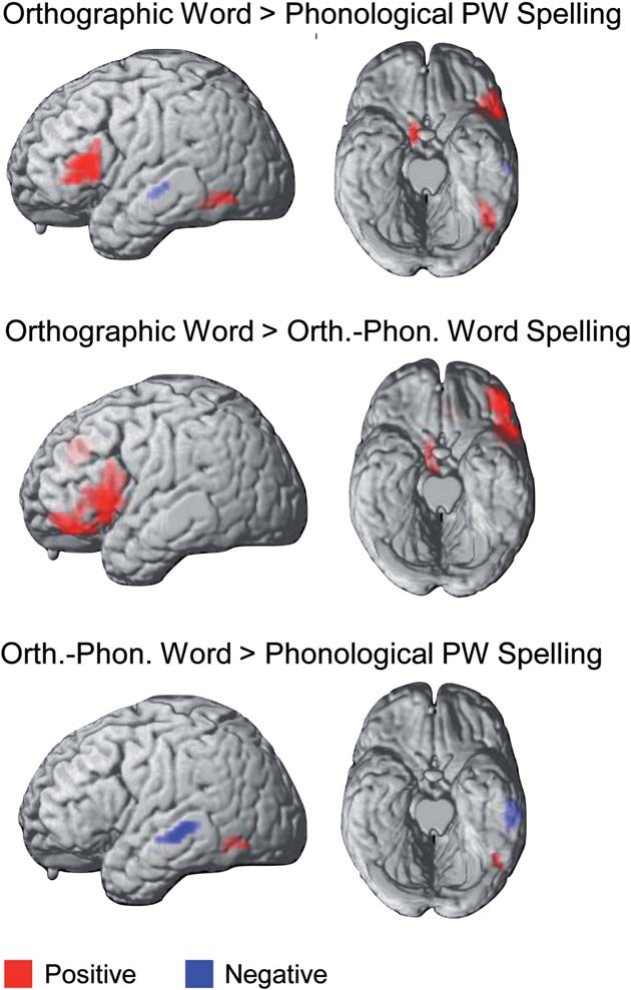
Contrasts among the spelling conditions. Activation maps for the contrasts between the orthographic, the orthographic‐phonological, and the phonological spelling condition. All comparisons are thresholded at *P* < 0.001, voxelwise, with a cluster extent threshold of *P* < 0.05 (FWE corrected).

For these comparisons we limited the search space to regions activated by the spelling conditions relative to the control conditions. The search mask was a combination of the activation maps identified by the contrasts in Table 4. A first finding—shown in Table 5 and Figure 3—was that the orthographic spelling condition elicited more activation than both the orthographic‐phonological and the phonological spelling condition in left IFG, medial superior frontal/paracingulate gyrus, and the right caudate.

**Table 5:** Contrasts among the spelling conditions

			MNI coordinates			
Region	H	k	*x*	*y*	*z*	*t*
*Orthographic word > phonological pseudoword spelling*						
Left inferior frontal gyrus (pTri / pOp)	L	127	−51	20	7	4.97
Superior frontal gyrus / Paracingulate gyrus	L	28	−9	47	28	4.02
Ventral occipitotemporal cortex	L	32	−45	−55	−11	5.13
Caudate	R	62	12	8	7	5.22
*Orthographic word > orthographic‐phonological word spelling*						
Left inferior frontal gyrus (pTri / pOp)	L	287	−48	20	7	6.32
Superior frontal gyrus / Paracingulate gyrus	L	87	−9	44	28	5.14
Caudate	R	55	12	8	7	4.24
*Orthographic‐phonological word > phonological pseudoword spelling*						
Ventral occipitotemporal cortex	L	12	**‐**45	**‐**58	**−**11	3.93
*Phonological pseudoword > orthographic* **‐** *phonological word spelling*						
Superior temporal gyrus	L	37	**−**54	**‐**16	**−**8	5.35
*Phonological pseudoword > orthographic* *word spelling*						
Superior temporal gyrus^a^	L	6	**−**57	−22	**−**5	3.73

Comparisons are thresholded at *P* < 0.001, voxelwise, with an cluster extent threshold of *P* < 0.05, FWE corrected. Abbreviations: pTri = pars triangularis; pOp = pars opercularis; H = Hemisphere; k = cluster extent in voxels.

a *P* < 0.001 (uncorrected)

For our hypotheses differences between the spelling conditions in left vOT were of main interest. Table 5 and Figure 3 show that higher activation for the orthographic than for the phonological spelling condition was found in a cluster with a peak in the ITG at MNI coordinates [−45 −55 −11]. A similar cluster was identified with higher activation for orthographic‐phonological than for phonological spelling with a peak at [−45 −58 −11]. Even with a more liberal threshold (*P* <  .001, uncorrected) no activation differences were identified between the word spelling conditions in these regions.

In left STG, the pseudoword spelling condition elicited higher activation than both the orthographic‐phonological and the orthographic condition. We want to note that the latter comparison was only significant at a lowered threshold (*P* < 0.001, uncorrected).

#### Region of Interest (ROI) Analysis

**Figure 4:**
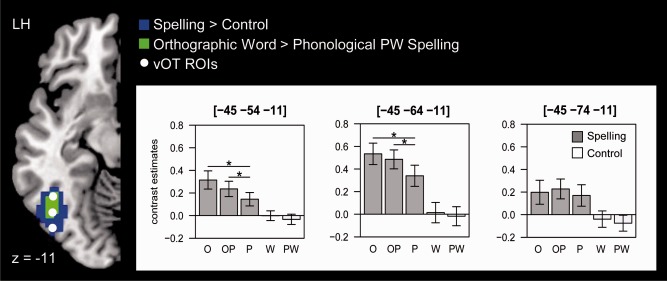
ROI analysis in left vOT. The left panel shows the orthographic > phonological spelling cluster (green) superimposed on the spelling > control cluster (blue) in left vOT as well as the approximate locations of the ROIs. Activation clusters are shown on an axial slice at *z* = −11. The right panel shows mean contrast estimates (vs. rest) for spheres (*r* = 4 mm) centered at the given coordinates. Error bars denote ±1 standard error of mean. Abbreviations: O = orthographic word spelling, OP = orthographic‐phonological word spelling, P = phonological pseudoword spelling, W = word control, PW = pseudoword control. [Color figure can be viewed in the online issue, which is available at http://wileyonlinelibrary.com.]

In addition to the whole‐brain analysis, we investigated differences between the spelling conditions in left vOT with a ROI‐based analysis. Of main interest was the posterior extent of the increased activation for the word spelling conditions relative to the pseudoword spelling condition identified at *y*= −55 (orthographic) and *y* = −58 (orthographic‐phonological). More specifically, we were interested in whether these peaks were different from the left vOT peak at *y* = −64 identified for all spelling conditions relative to the control conditions. For this, we computed a spelling > control contrast (collapsed across word and pseudoword conditions). Figure 4 illustrates the resulting left vOT cluster in relation to the orthographic word > phonological pseudoword spelling cluster. Within this spelling > control cluster, we selected three ROIs varying along the anterior–posterior axis. The middle ROI corresponded to the cluster peak at MNI coordinates [−45 −64 −11]. We further selected a 10 mm more anterior ROI (*y* = −54) and a 10 mm more posterior ROI (*y* = −74) to cover the entire anterior‐to‐posterior extent of the cluster. The x and z coordinates for these ROIs were kept constant. Figure 4 shows the approximate locations of the ROIs. Mean contrast estimates for each condition versus rest were then extracted for spheres (*r* = 4 mm) centered at the mentioned coordinates.

The main finding of the ROI analysis was that the activation pattern of higher activation for both word spelling conditions relative to the pseudoword spelling condition found in the anterior ROI at *y* = −54 was also present in the middle ROI at *y* = −64 (*p*s < 0.05, Bonferroni corrected). As can be seen in Figure 4, this pattern was no longer present in the posterior ROI in which the spelling conditions did not differ from each other. In addition, we want to note that the activation levels for the spelling conditions in the middle vOT ROI (at the spelling > control peak) were nearly twice as high as in the anterior and posterior vOT ROIs.

## DISCUSSION

The present fMRI relied on a spelling task to investigate the hypothesis that left vOT serves as memory store for known written words and thereby functions as the orthographic word lexicon assumed by dual‐route theories of reading and spelling [e.g., Coltheart et al., 2001]. For this, the orthographic word spelling condition of the present study was of critical importance as correct spelling decisions had to be based on orthographic whole‐word representations. Such enforced reliance on orthographic word memories was not necessarily given in the orthographic‐phonological word spelling condition and no reliance on such representations was possible in the phonological spelling condition that presented spoken pseudowords. Our main findings were that both word spelling conditions elicited higher left vOT activation than the pseudoword spelling condition which in turn elicited higher activation than the word spelling conditions in left superior temporal regions. In addition, the conflict‐resolution demands of the orthographic word spelling condition led to higher left frontal activation for ths condition relative to the orthographic‐phonological word condition in which no conflict existed. Importantly, however, this was not accompanied by a difference in left vOT activation.

### The Role of Left vOT in Spelling

The finding of pronounced left vOT activation for the orthographic word spelling condition relative to the control condition speaks for the view that activation of left vOT during spelling can be attributed to the retrieval of orthographic whole‐word representations. The position that the left vOT serves as memory store for the spellings of known words also finds support in recent neuroimaging studies of spelling showing that left vOT is sensitive to lexical factors such as word frequency [Rapp and Lipka, 2011; Rapp and Dufor, 2011]. In addition, the absence of a difference between the orthographic and the orthographic‐phonological word spelling condition in left vOT speaks against the concern that the conflict between orthographic and phonological decision tendencies in the orthographic spelling condition affected left vOT activation.

The findings for the phonological pseudoword spelling condition are somewhat unexpected. As mentioned in the Introduction, one expectation was that pseudoword spelling would result in similar or even higher left vOT activation compared to word spelling. This was based on the assumption that our spelling findings would correspond to findings from reading showing that the left vOT is involved in both whole‐word and sublexical coding [e.g., Schurz et al., 2010]. However, the reduced vOT activation for the phonological pseudoword spelling condition relative to both word spelling conditions speaks against a general correspondence of spelling and reading findings in left vOT.

In the Introduction, we also considered the possibility that pseudoword spelling may not activate left vOT. This was based on neuroimaging studies showing that sublexical spelling primarily relies on left STG and IFG rather than vOT [Omura et al., 2004; Beeson and Rapcsak, 2003] as well as neuropsychological studies showing that damage to left vOT does not affect sublexical spelling processes [Henry et al., 2007; Philipose et al., 2007]. However, in contrast to this expectation, we did observe activation in left vOT for the phonological pseudoword spelling condition relative to the control condition. A plausible account for this finding is offered by studies showing that spelling of pseudowords is strongly influenced by orthographic representations of phonologically similar words [Tainturier et al., 2013]. Accordingly, the auditory pseudowords of the phonological spelling condition may not only have elicited sublexical but also lexical processes. Specifically, the auditory pseudowords may have activated the representations of phonologically similar words, which in turn (automatically) activated their corresponding orthographic word representations. Such indirectly activated orthographic word representations may explain the observed vOT activation in the phonological pseudoword spelling condition.

The vOT activation peak at MNI coordinates [‐45 ‐64 ‐11] identified for all spelling conditions relative to the control conditions closely corresponds to the left vOT clusters found by previous neuroimaging studies of spelling, especially to activation peaks of the recent spelling meta‐analyses by Planton et al. [2013] at [−46 −62 −12] and by Purcell et al. [2011a] at [−52 −58 −16]. Although the peaks of the word > pseudoword spelling contrasts were localized more anterior (at [−45 −55 −11] for the orthographic and at [−45 −58 −11] for the orthographic‐phonological word spelling condition) the ROI analysis showed that higher activation for word compared to pseudoword spelling was also present at the general spelling > control peak at *y* = −64 (see Fig. 4). This suggests that both coordinates may be part of a functionally homogenous brain region, which we interpret as being involved in the retrieval of orthographic whole‐word representations during spelling. The ROI analysis further showed that the word > pseudoword spelling pattern found in the anterior and middle vOT ROI was no longer present in the posterior ROI at *y* = −74. One may speculate that the absence of a difference between word and pseudoword spelling potentially points to smaller orthographic codes (i.e., letters or letter sequences) in the posterior part of vOT. This would be consistent with accounts suggesting increasingly larger orthographic codes along the left ventral visual pathway [Dehaene et al., 2005; Vinckier et al., 2007]. It is also of interest that the present spelling activations did not extend to more anterior regions (beyond *y* = −49) which have been associated with amodal language processes [Lüders et al., 1991], semantic processing [e.g., Binder et al., 2009] or with mediating processes between orthography and semantics [Purcell et al., 2014].

Most of the neuroimaging research on orthographic processing in left vOT cortex is based on reading or reading‐related tasks which present visual letter strings as stimuli (e.g., lexical decision). The present findings add to the more limited set of studies, which show that the left vOT is responsive to auditory words in the context of spelling/writing tasks. If both visual word stimuli (in the context of reading) and auditory word stimuli (in the context of spelling) rely on the same orthographic whole‐word representations, then spatial overlap between reading and spelling activations in vOT is expected [Hillis and Rapp, 2004]. The present study cannot directly examine this expectation. However, the localization of the vOT clusters identified here by the spelling > control and by the orthographic > phonological spelling contrast correspond to left vOT clusters reliably identified in neuroimaging studies of word reading [e.g., Mechelli et al., 2003; Turkeltaub et al., 2002] and, importantly, to clusters identified in our previous studies on orthographic familiarity effects (i.e., high‐frequency < low‐frequency words and words < pseudohomophones and pseudowords) in mid to posterior left vOT [Kronbichler et al., 2007, 2004; Ludersdorfer et al., 2013]. As mentioned in the Introduction, two recent studies also provided direct evidence for an overlap between word reading and spelling activation in left vOT [Rapp and Lipka, 2011; Purcell et al., 2011b].

The present findings cannot speak to the nature of orthographic word representations in left vOT. A prominent assumption is that visual letter information is lost along the left ventral visual pathway resulting in abstract orthographic representations in the most anterior parts [Dehaene et al., 2005]. Recent evidence even suggests that a region corresponding to the classic localization of the VWFA at around *y* = −58 [Cohen et al., 2002] hosts modality‐independent rather than visual representations. Two fMRI studies demonstrated activation in this region for congenitally blind participants during braille reading [Reich et al., 2011] or “reading” of soundscapes [Striem‐Amit et al., 2012]. Accordingly, the region was characterized as meta‐modal reading area [Dehaene and Cohen, 2011]. However, it may be premature to generalize from findings with congenitally blind readers to sighted readers. In a recent study, we provided evidence suggesting that—in sighted participants—the vOT region corresponding to the VWFA exhibits an activation profile typical for a visual rather than a meta‐modal region [Ludersdorfer et al., 2013]. In a one‐back task, we found this region (identified by an orthographic familiarity effect with visual words < pseudowords) to exhibit high activation to visual artificial word‐like stimuli (i.e., false fonts) and marked deactivation to auditory artificial word‐like stimuli (i.e., reversed speech). According to the phenomenon of cross‐sensory suppression [Laurienti et al., 2002] such deactivation during the processing of demanding auditory stimuli is expected in (modality‐specific) visual regions. The similar localization of the present spelling cluster and the cluster found in Ludersdorfer et al. [2013] tentatively suggests that the orthographic representations accessed in the present spelling study were not fully abstract.

### Frontal Regions Engaged by Spelling

With respect to frontal regions, recent neuroimaging studies of spelling [Rapp and Dufor, 2011; Rapp and Lipka, 2011] suggested that the left inferior frontal junction (IFJ) at the border between IFG pOp and precentral gyrus is critically engaged by spelling processes. The IFJ is assumed to be involved in the selection of orthographic word representations from orthographic long‐term memory (assumed to be stored in left vOT) and in the resolution of uncertainties about the correct spelling. This interpretation is based on the more general account that the left IFJ is involved in cognitive control processes, specifically in the resolution of conflicting response options as in the Stroop task [e.g., Brass et al., 2005].

The present frontal activation findings are only partly consistent with previous spelling findings. We found widespread left lateral frontal activations for all three spelling conditions not only restricted to left IFJ but encompassing IFG, precentral gyrus and the insula as well as medial frontal activations in anterior cingulate cortex and paracingulate gyrus. In addition, the main peaks of the left lateral frontal clusters for the word spelling conditions (orthographic and orthographic‐phonological) did not correspond to the IFJ and were located more anterior and more ventral in the insula. Only the main peak of the comparison of the phonological pseudoword spelling condition to the control condition was located in precentral regions corresponding to the IFJ. However, these frontal activations may not reflect spelling‐specific processes. Instead, they may only reflect the higher cognitive demands of the spelling conditions compared to the control conditions as reflected in prolonged response latencies and higher proportions of erroneous responses. This interpretation is in line with findings of Binder et al. [2004] showing activation levels in these frontal regions correlate with time on task and task difficulty.

In addition, the left IFG (including IFJ) and superior frontal/paracingulate gyrus exhibited higher activation for the orthographic relative to both other spelling conditions. Such an activation increase for the orthographic spelling condition was expected in regions associated with conflict resolution as well as decision and response selection [Brass et al., 2005; Botvinick et al., 1999] due to the conflict between orthographic and phonological spelling decision tendencies.

### Left STG in Spelling

In left posterior STG, we observed higher activation for phonological pseudoword spelling relative to both word spelling conditions. This is consistent with previous neuroimaging studies showing that sublexical spelling via phoneme–grapheme conversions primarily relies on left STG and IFG [Beeson and Rapcsak, 2003; Omura et al., 2004]. More specifically, the increased STG activation in the present study may reflect the increased phonological processing demands required for sublexical phoneme–grapheme conversions. In line with this interpretation, the location of the present STG activations closely corresponds to the localization of a phonetic/phonological analysis cluster of a recent fMRI meta‐analysis of sublexical speech perception components [Turkeltaub and Coslett, 2010]. However, a different explanation can be drawn when considering the behavioral results of the spelling conditions. Here, both the orthographic and the phonological spelling condition led to more errors than the orthographic‐phonological condition. While the increase in errors in the orthographic condition is likely caused by the demands of conflict resolution the increase in errors in the phonological spelling condition may have resulted from a higher perceptual difficulty posed by pseudowords in the noisy environment of the scanner. Different to the perception of words pseudowords cannot benefit from top‐down constraints from phonological word representations to compensate for insufficient sensory input. Thus, the higher STG activation for the phonological spelling condition might also be explained by the higher perceptual difficulty of pseudowords.

Interestingly, the present study failed to identify STG activation for the word spelling conditions relative to the word control condition. This stands in contrast to previous studies as the spelling meta‐analysis by Purcell et al [2011a] identified bilateral STG activations and the study by Rapp and Lipka [2011]—which used the same spelling task as the present study—found left STG more activated for word spelling compared to an auditory control task. Absence of STG activations for the present word spelling conditions may be due to the gender decision task of our word control condition. Similar to the present spelling task, the gender decision task also required attention to the auditory stimulus. The control task of Rapp and Lipka [2011], however, may have distracted attention away from the auditory words as only the case format of the accompanying probe letters had to be evaluated. Hence, while STG activation reflecting auditory (word) processing was equal for spelling and control task in the present study such activation was presumably reduced for the control task (relative to the spelling task) in the study of Rapp and Lipka. Of further interest is that in other spelling studies—contributing to the STG cluster in the meta‐analysis of Purcell et al. [2011a]—word spelling was contrasted with a low‐level auditory control condition (i.e., pure tone processing).

### Occipito‐Parietal Cortex in Spelling

Similar to the spelling meta‐analysis by Purcell et al. [2011a], the present study did identify an occipitoparietal cluster for the word spelling conditions. Purcell et al. interpreted this region to be involved in orthographic working memory (i.e., graphemic buffer). Interestingly, the presently found cluster was situated posterior to the angular gyrus on the border between lateral occipital gyrus and superior parietal lobule. This failure to identify the angular gyrus in the present spelling study is interesting because of the prominent role attributed to the angular gyrus in the neuropsychological study of reading and spelling impairments [Dejerine, 1892]. Purcell et al. [2011a] provide an extensive discussion of the difference between older neuropsychological research and recent neuroimaging studies that mostly failed to identify angular gyrus activation. One possible explanation is based on the view that the angular gyrus is involved in conceptual and semantic processing [e.g., Binder et al., 2009]. Since spelling (and reading) tasks are not specifically designed to engage semantic processes, they may not elicit reliable activation in semantic regions [Purcell et al., 2011a].

## CONCLUSION

The present fMRI study provides support for the position that the left vOT hosts neuronal representations coding for the exact letter strings of all known words. The assumption of such an orthographic word lexicon is controversial, but constitutes an essential component of cognitive dual‐route models of reading and spelling [e.g., Coltheart, 2004]. Our study used a spelling task in which, on every trial, participants had to decide whether a visually presented letter was present in the written form of an auditorily presented word. In a critical condition, we ascertained that correct spelling decisions could only be based on orthographic whole‐word representations. We identified a left vOT cluster with higher activation for this orthographic word spelling condition relative to a control condition (controlling for auditory input and motor output) and to a pseudoword spelling condition in which reliance on orthographic representations was not possible and decisions had to be based on phoneme‐letter associations. The location of this orthographic spelling cluster corresponds to the left vOT region typically found to be engaged by visual word reading. These results support the position that left vOT may represent the neuronal equivalent of the cognitive orthographic word lexicon.

## Supporting information

Supplementary Information
