# Supplementary material for: Accessing orthographic representations from speech: The role of left ventral occipitotemporal cortex in spelling
Source: Hum Brain Mapp. 2014 Dec 12;36(4):1393–406. doi: 10.1002/hbm.22709 (PMC4383651; doi:10.1002/hbm.22709)
Supplement: Supplementary file 1 — Supplementary Information [file HBM-36-1393-s001.doc]

**SUPPLEMENTARY RESULTS**

**Accessing Orthographic Representations from Speech: The Role of Left Ventral Occipitotemporal Cortex in Spelling**

Philipp Ludersdorfer^1,2^, Martin Kronbichler^1,2,3^, & Heinz Wimmer^1,2^

^1^Centre for Cognitive Neuroscience, University of Salzburg, Austria

^2^Department of Psychology, University of Salzburg, Austria

^3^Neuroscience Institute, Christian-Doppler-Clinic, Paracelsus Medical University Salzburg, Austria

**Spelling conditions versus rest**

*Figure S1.* Spelling conditions versus rest. Brain regions activated by the orthographic, the orthographic-phonological, or the phonological spelling condition compared to rest. Colors denote height of
*t* statistic. All comparisons are thresholded at *p* < .001, voxelwise with an additional cluster extent threshold of *p* < .05, FWE corrected.

Table S1.

*Spelling conditions versus rest. Brain regions more activated by the orthographic, orthographic-phonological or phonological spelling condition compared to rest (voxelwise threshold: p < .001, cluster extent threshold:
p < .05, FWE corrected).*

| Region | H | k | MNI coordinates | | | t |
| --- | --- | --- | --- | --- | --- | --- |
|  |  |  | x | y | z |  |
|  |  |  |  |  |  |  |
| *ORTHOGRAPHIC (WORD) SPELLING* | | | | | | |
| Superior Temporal Gyrus (posterior) | L | 6425 | -54 | -19 | 1 | 16.02 |
| Precentral Gyrus |  |  | -48 | 5 | 16 | 14.18 |
| Superior Temporal Gyrus (posterior) | R | 1218 | 60 | -25 | 1 | 15.22 |
| Precentral Gyrus | R | 183 | 51 | -1 | 43 | 5.77 |
| Supramarginal Gyrus | R | 140 | 45 | -43 | 43 | 6.27 |
| Ventral occipitotemporal cortex | L | 94 | -45 | -64 | -11 | 6.89 |
| Pallidum | R | 179 | 15 | 2 | -2 | 5.58 |
|  |  |  |  |  |  |  |
| ORTHOGRAPHIC-PHONOLOGICAL (WORD) SPELLING | | | | | | |
| Superior Temporal Gyrus (posterior) | L | 5207 | -51 | -19 | 1 | 16.03 |
| Precentral Gyrus |  |  | -48 | 5 | 16 | 12.89 |
| Superior Temporal Gyrus (posterior) | R | 1174 | 60 | -25 | 1 | 15.28 |
| Precentral Gyrus | R | 183 | 51 | -1 | 43 | 5.45 |
| Supramarginal Gyrus | R | 101 | 45 | -37 | 37 | 5.78 |
| Ventral occipitotemporal cortex | L | 69 | -45 | -64 | -11 | 6.30 |
| Cerebellum | R | 421 | 30 | -64 | -26 | 9.29 |
|  |  |  |  |  |  |  |
| PHONOLOGICAL (PSEUDOWORD) SPELLING |  |  |  |  |  |  |
| Superior Temporal Gyrus (posterior) | L | 5581 | -54 | -16 | -2 | 17.77 |
| Precentral Gyrus |  |  | -51 | 5 | 16 | 13.61 |
| Superior Temporal Gyrus (posterior) | R | 1281 | 60 | -25 | 1 | 16.49 |
| Precentral Gyrus | R | 93 | 51 | -1 | 43 | 6.15 |
| Middle Frontal Gyrus | R | 61 | 45 | 29 | 28 | 4.75 |
| Supramarginal Gyrus | R | 168 | 45 | -43 | 43 | 6.14 |
| Ventral occipitotemporal cortex* | L | 19 | -45 | -64 | -11 | 4.38 |
| Cerebellum | R | 297 | 30 | -64 | -26 | 9.47 |
|  |  |  |  |  |  |  |

* This cluster did not survive the cluster extent threshold of p < .05 (FWE corrected).
